# Private provider participation in statewide immunization registries

**DOI:** 10.1186/1471-2458-6-33

**Published:** 2006-02-15

**Authors:** Sarah J Clark, Anne E Cowan, Diana L Bartlett

**Affiliations:** 1 Child Health Evaluation and Research Unit, Division of General Pediatrics, University of Michigan, 300 North Ingalls Rm 6E06, Ann Arbor, Michigan, 48109-0456, USA; 2 Immunization Services Division, National Immunization Program, Centers for Disease Control

## Abstract

**Background:**

Population-based registries have been promoted as an effective method to improve childhood immunization rates, yet rates of registry participation in the private sector are low. We sought to describe, through a national overview, the perspectives of childhood immunization providers in private practice regarding factors associated with participation or non-participation in immunization registries.

**Methods:**

Two mailed surveys, one for 264 private practices identified as registry non-participants and the other for 971 identified as registry participants, from 15 of the 31 states with population-based statewide immunization registries. Frequency distributions were calculated separately for non-participants and participants regarding the physician-reported factors that influenced decisions related to registry participation. Pearson chi-square tests of independence were used to assess associations among categorical variables.

**Results:**

Overall response rate was 62% (N = 756). Among non-participants, easy access to records of vaccines provided at other sites (N = 101, 68%) and printable immunization records (N = 82, 55%) were most often cited as "very important" potential benefits of a registry, while the most commonly cited barriers to participation were too much cost/staff time (N = 36, 38%) and that the practice has its own system for recording and monitoring immunizations (N = 35, 37%). Among registry participants, most reported using the registry to input data on vaccines administered (N = 326, 87%) and to review immunization records of individual patients (N = 302, 81%). A minority reported using it to assess their practice's immunization coverage (N = 110, 29%) or generate reminder/recall notices (N = 54, 14%). Few participants reported experiencing "significant" problems with the registry; the most often cited was cost/staff time to use the registry (N = 71, 20%).

**Conclusion:**

Most registry participants report active participation with few problems. The problems they report are generally consistent with the barriers anticipated by non-participants, but did not impede participation. Recruitment efforts should focus on demonstrating the benefits of the registry to providers. In addition, many participants are not utilizing the full range of registry features; further study is needed to determine how best to increase use of these features.

## Background

Immunization registries are defined as confidential, population-based computerized systems that contain information regarding children's vaccinations [1]. The success of immunization registries is dependent on broad participation of immunization providers and comprehensive enrollment of persons vaccinated. Provider participation is critical for achieving the Healthy People 2010 objective of increasing to 95% the proportion of children less than 6 years of age with two or more vaccinations recorded in fully operational population-based immunization registries [2]. However, a CDC survey of its 56 immunization grantees (50 states, 5 cities, District of Columbia) showed that at the end of 2004 only 39% of private immunization providers were actively submitting data to a statewide or regional registry and only 48% of children 0–6 years of age were enrolled in a statewide or regional registry [3].

In considering strategies to increase private provider participation, it is important to take into account both the barriers perceived by non-participants and the experiences of current registry participants. The assessments of provider perspectives documented in the published literature [4-7] reflect only single states or urban areas; lacking is a perspective on provider participation across a wider sector of registry participants and non-participants, which may provide some valuable "lessons learned" for those seeking to improve provider participation rates. Therefore, this study was designed to give a national overview of the perspectives of childhood immunization providers in private practice regarding participation in immunization registries.

## Methods

### Selection of survey states

Of the 31 states that reported having a population-based, statewide immunization registry in 2001, officials from the immunization registry support branch of the Centers for Disease Control and Prevention (CDC) selected 15 states: AZ, AR, DE, ID, MI, MO, MS, ND, NV, OH, OR, TX, UT, WI, and WV. These states were systematically chosen to represent variation in population, US region, estimated level of private provider registry participation, and existence of state-level immunization registry reporting mandates. (At the time of the survey, such legislative mandates existed in AR, AZ, DE, MD, MI, MS, and TX.) In addition, immunization program officials from these states agreed to provide information for generating the survey sample.

### Survey sample

The survey sample was based on practice sites participating in the federal Vaccines for Children (VFC) program through the immunization programs of the 15 study states. Because the study focus was private physician practices that provide primary care in an outpatient setting, we excluded other types of immunization providers (e.g., public health departments, schools).

For purposes of classifying registry participation, we asked immunization officials in the 15 study states to provide contact information for: (1) practices enrolled in the VFC program and (2) practices enrolled in the state immunization registry. We then matched private VFC sites with practices on the registry list. Practices on the VFC list that did not appear on the registry list were assumed to be registry non-participants. Five states (AR, DE, MS, ND, UT) reported that all VFC practices also participate in the registry; therefore, all practices were designated as registry participants. One state (NV) did not have a separate registry list available, so we chose to treat all practices as registry participants.

With the goal of having approximately 1,200 practices in the overall sample, we randomly selected a sample of practices, with a target of 75–90 practices per state, for each of the 15 states. Within each state, the selection of practices was roughly proportional to that state's balance of registry non-participants and participants. As the study did not attempt to produce national point estimates, a weighted sampling scheme was not employed. The final sample contained 1,235 practices: 264 classified as registry non-participants and 971 as registry participants. For survey mailing purposes, a single physician was identified to be the respondent for each practice; typically, this was the individual listed as either the VFC or the registry contact physician.

### Survey design and implementation

We designed two brief surveys, one for registry non-participants and one for participants. Both surveys included questions about verification of participation status, suggestions for enhancing private provider participation, and respondent specialty and practice characteristics. The registry non-participant survey also included questions on barriers to participation; perceived importance of potential benefits of a registry; factors essential for participation; and likelihood of future participation. The registry participant survey included questions about influences on practice's decision to participate in the registry; ways in which the practice interacts with and uses the registry; and extent of any problems experienced with the registry. Survey cover letters were individualized to each contact physician, while the survey instrument was customized with the specific name of each state's registry. Survey questions were pilot-tested with a convenience sample of physicians in Michigan to ensure clarity and ease of administration. The survey instruments are found in the Appendix [see Additional File-1].

Surveys were fielded in two phases, corresponding to the availability of state-supplied VFC and registry lists. The first phase (AZ, AR, ID, MI, MS, NV, TX) occurred between August-December 2002; the second phase (DE, MO, ND, OH, OR, UT, WI, WV) was conducted between February-June 2004. After an initial mailing to each practice, two subsequent mailings were sent to non-respondents. The study protocol was approved by the Institutional Review Boards of the University of Michigan Medical Center and the CDC.

### Data analysis

We generated frequencies for responses to all survey items. We then used Pearson chi-square tests of independence to test for association among categorical variables. P-values less than 0.05 were considered significant. All analyses were conducted using SAS version 8.2 (SAS Inc., Cary, NC).

## Results

Of the 1,235 practices selected, 13 were excluded because mailing materials were returned as undeliverable. From the remaining 1,222 practices, we received 756 surveys eligible for data analysis, for a response rate of 62%. Respondent characteristics are shown in Table 1; participants differed from non-participants in the mix of practice ownership/affiliation.

### Accuracy of registry participation classification

Of the 152 practices initially classified from state-supplied data as registry non-participants, 62% (N = 95) confirmed that their practice does not participate in their state immunization registry, 29% (N = 44) said that their practice actually does participate in the registry, and 9% (N = 13) were unsure. Of the 604 practices initially classified as registry participants, 62% (N = 374) confirmed that their practice currently participates in their state immunization registry; 33% (N = 202) said that their practice does not participate and 5% (N = 28) were unsure.

### Perspectives of registry non-participants

Among the 95 confirmed non-participants, 44 (51%) reported that their practice had not been contacted about participating in the registry, while 15 (18%) were unsure. Of the 27 (31%) who reported that their practice had been contacted about registry participation, almost all (N = 24, 89%) were contacted by state immunization program staff, one (4%) by a managed care organization, one (4%) by a professional association, and one (4%) by another party.

As shown in Table 2, the most common reasons given for not participating in the registry were that participation requires too much cost/staff time and that the practice has its own system for recording and monitoring immunizations.

Factors most frequently cited by non-participants as being "essential to participation" included compatibility of registry technology and office computers (N = 55, 58%), automated data entry (N = 44, 46%), and on-site technical assistance from registry staff (N = 31, 33%). Less common factors were: increased participation from other providers in the community (N = 28, 29%); legal advice or expertise to address confidentiality concerns (N = 25, 26%); support for the registry from the state medical or specialty society (N = 14, 15%); and support from the practice's nursing or administrative staff (N = 13, 14%).

As a general gauge of non-participant interest in the registry, confirmed non-participants were asked the likelihood that their practice would participate in the registry in the next two years. Fourteen (17%) indicated that they were likely to participate, while an additional 23 (28%) indicated it was possible that they would participate. Of the remainder, 27 (33%) indicated that it was unlikely that they would participate in the next two years and 18 (22%) reported they were "not at all likely" to participate. Reported likelihood of participating did not differ by existence of a state registry reporting mandate.

All respondents (N = 152) to the non-participant survey were asked the importance of potential benefits of an immunization registry. The majority of respondents cited as "very important" easy access to records of vaccines provided at other sites (N = 101, 68%) and printable immunization records (N = 82, 55%). Less frequently cited as "very important" were: ability to assess practice's immunization coverage rate (N = 64, 43%); ability to general reminder/recall notices (N = 62, 42%); and ability to document vaccines given for HEDIS/managed care assessments (N = 45, 31%).

### Perspectives of registry participants

Among the 374 confirmed registry participants, 184 (50%) had been participating in the registry for at least 3 years, 118 (32%) for 1–2 years, and 51 (14%) for less than one year; 14 (4%) were unsure. Most participants reported using the registry to input data on vaccines administered (N = 326, 87%) and to review immunization records of individual patients (N = 302, 81%). The majority also print immunization records for patients from the registry (N = 225, 60%). Few reported using the registry to assess immunization coverage for the practice (N = 110, 29%) or generate reminder/recall notices (N = 54, 14%). Eleven (3%) reported no active uses (i.e., enrolled but not actively interacting with the registry).

Among practices that report immunization data to the registry, the staff responsible for doing so are typically nurses (N = 299, 92%), and less often clerical or billing staff (N = 87, 27%) or other personnel (N = 17, 5%). With regard to the amount of time spent on this task, 151 respondents (41%) estimated less than 2 hours/week, 124 (34%) estimated 2–5 hours/week, and 45 (12%) said more than 5 hours/week; 45 (12%) were unsure. The more vaccines administered to children in a typical week, the more hours/week staff spent reporting data to the registry (p < 0.0001). Almost half of respondents (N = 176, 48%) interact with the registry by internet connection, 133 (36%) submit hard-copy data by mail or fax, and 69 (19%) interact with the registry by modem; 35 (10%) of respondents reported more than one mode of interaction.

In recalling influences on their practice's decision to participate in the registry (Table 3), respondents most often cited the need to consolidate records for patients who receive vaccines at multiple sites and, when present, state mandates for participation.

Most respondents did not report significant problems with their state immunization registry. As shown in Table 4, none of the list of concerns was cited as a "significant problem" for more than 20% of respondents. Having "significant" problems with the cost/staff time was associated with greater staff time spent reporting data (p < .001), and using non-internet methods of registry interaction (p = .02). Reporting "significant" problems with data accuracy/completeness was not associated with reported uses of the registry.

All respondents to the participant survey (N = 604) were asked an open-ended question on the most important thing that registry officials could do to enhance provider participation in the registry. Of the 362 that responded (60%), 84 (23%) suggested specific refinements, such as addressing login problems, entering legacy data, or enhancing registry functions; 79 (22%) urged more education about the registry and its benefits to providers, office staff, and/or parents. Sixty-eight (19%) mentioned simplification of systems, such as automating data input; 47 (13%) suggested greater compatibility with office computers and systems; and 44 (12%) suggested more technical assistance and training; the remainder had other, miscellaneous comments.

## Discussion

This study presents, from the practitioner's perspective, the experiences and attitudes that influence private provider participation in state immunization registries. Non-participants noted that easy access to records of vaccines administered at other sites of care, as well as the ability to generate printable immunization records, were very important potential registry benefits; however, many non-participants felt their practice had an adequate system for recording immunizations, and many expressed concerns about the costs of registry participation. Most registry participants used the registry to review immunization records for patients, but used the practice assessment and recall features less commonly. Cost concerns were noted, but with less frequency than non-participants. Participants offered numerous suggestions on how to improve private provider participation in their state's registry.

This study was designed to present a national overview of the perspectives of private immunization providers on statewide immunization registries. However, there are important challenges to such an assessment. Not every state has a population-based, statewide immunization registry. For those that do, each state registry is at a different phase of development; each has different features, functionality, and level of automation. Moreover, registry-related policies vary from state to state, from reporting mandates to incentives for participation. While this study was not intended to produce state-level results, we cannot overlook the fact that there is tremendous variability from state to state on most aspects of registries [3]. This assessment, therefore, represents an amalgamation of experiences across 15 states.

The effort to recruit immunization providers into an immunization registry begins with identifying the potential pool of participants. The sample for this study was based on the group of childhood immunization providers most likely targeted for registry participation – those participating in the federal Vaccines for Children (VFC) program. Because VFC is administered through state immunization programs, practices participating in VFC would already have some level of working relationship with state officials surrounding childhood immunizations. We compared VFC and registry participation lists to perform initial classifications of participation status; this initial status was confirmed by 62% of respondents to both the non-participant and participant surveys. The reasons for misclassification may be different for the two groups. With regard to those initially classified as non-participants, misclassification may reflect outdated participant listings or recent status changes, particularly in states that were actively recruiting private physicians. For those initially classified as participants, misclassification may be a function of how states define registry participation; as noted in Methods, five states reported that all VFC practices are registry participants, even though they may not have been actively submitting data or using the registry.

A common reason given by respondents for not participating was having an existing system for recording and monitoring immunizations. Certainly, practice-based and even regional systems for tracking childhood immunizations exist; however, these systems do not have the breadth of a statewide registry. In recruitment efforts with private practices, registry officials may need to provide data on the proportion of children who receive vaccines at multiple sites, so that providers can appreciate the benefits of participating in a statewide system. Among participants, consolidating records was the factor most commonly cited as being very influential to their participation decision. Therefore, consolidation of records should be (or continue to be) a strong marketing point when recruiting providers. At the same time, registry officials must continue to explore linkages with billing and patient management systems, so that participation does not engender a substantial burden of cost and/or staff time.

Barriers reported by non-participants were relatively consistent with the problems experienced by participants, most commonly cost/staff time associated with using the registry. However, findings from this study suggest that non-participants' perceptions of potential problems exceed the extent of problems actually experienced by participants. It seems that, while these problems may not go away completely, they generally do not inhibit participation. Registry recruitment and marketing efforts must emphasize that problems can be (and have been) overcome; concrete examples and peer assistance from staff in currently participating practices might prove helpful in this regard.

Findings also suggest that problems with the cost/staff time associated with inputting data to the registry were mitigated by internet access to the registry. However, more than a third of participants reported sending hard-copy data by phone or fax. Registry officials may need recruiting strategies specific to those practices that do not already have an internet connection and may be hesitant or unwilling to establish and pay for internet access.

It is interesting to note that non-participants' reasons for not participating did not necessarily correspond to their factors essential for participation; this suggests that recruitment should focus on what will get providers on board, rather than trying to ameliorate perceived barriers. Registry officials should seek input from physicians as they develop and enhance their registries to ensure that the factors physicians believe will influence their participation are being addressed to the extent possible. In an open-ended question on this survey, registry participants offered numerous targeted suggestions for improving registry functionality, many addressing technical features. Participants also suggested further educational efforts, directed to office staff and parents, as well as providers.

Data from non-participants indicate a strong potential for growth. Only half recalled being contacted about registry participation, and almost a third gave as the reason for not participating that they have not heard about it. Many appeared to be open to possible participation in the next 2 years. As individual states roll out their private provider recruitment efforts, it can be expected that a considerable proportion of private practices will become participants. Unfortunately, given the current number of functioning statewide registries, even an enthusiastic response to provider recruitment efforts will not be sufficient to achieve the Healthy People 2010 goals for registry participation nationally [2].

We found a high level of "any use" among registry participants. However, relatively few participants are utilizing more enhanced features of their registry, such as generating reminder/recall notices, a proven strategy to improve immunization coverage rates [8,9]. The registries in all but one of the 15 study states had reminder/recall functionality at the time of the survey (CDC, unpublished data). Further study should assess how best to expand the use of registry-based reminder/recall and other enhanced registry features.

### Limitations

There are several limitations to this study. First, as in any mailed survey, there is the possibility of response bias. However, the response rate is consistent with that of other published studies of physician practice patterns [10]. Second, the study was designed to provide a national overview only; the sample sizes in each state were not designed to generate state-level estimates nor was the study designed to provide statistically representative national estimates. Moreover, given the state-to-state variation in registry functionality and development, we limited the amount and type of bivariate analyses, so that we did not misattribute state-to-state variation to other factors. Third, the sample was limited to VFC providers; results may not be generalizable to non-VFC providers. However, the VFC provider population is a logical target for initial immunization registry recruitment efforts, so the findings are valuable in this respect. Finally, the survey was intended to collect information on physician perspectives of immunization registries. Information from other types of providers (e.g., nurses) will offer additional, valuable perspectives.

## Conclusion

This survey provides an overview of the perspective of both participating and non-participating private providers on statewide immunization registries. As would be expected, non-participants want a registry system that is simple and does not require a substantial amount of money or staff time. In subsequent recruitment efforts, state registry officials should highlight positive experiences by current participants – including strategies to overcome time and cost barriers – as a recruiting tool.

Communication between registry officials and physician practices does not stop with their agreement to participate. Just as registry development is continuous and dynamic, registry officials must continue to educate and train participants, as well as solicit their feedback. Many registry participants who responded to this survey suggested follow-up training, as well as improved technical features, as ways to enhance the functionality and usefulness of the registry in everyday clinical practice.

## Competing interests

The author(s) declare that they have no competing interests.

## Authors' contributions

SJC was responsible for overall study design, instrument development, and data interpretation. AEC participated in instrument development, data collection, and manuscript preparation. DLB participated in data interpretation and critical review of the manuscript. All authors read and approved the final manuscript.

## Pre-publication history

The pre-publication history for this paper can be accessed here:



## Supplementary Material

Additional File 1 Templates of registry participant and registry non-participant survey instruments. Click here for file

## Figures and Tables

**Table 1:** Characteristics of Survey Respondents by Initial Registry Participation Classification

	**Participant Survey (N = 604)**		**Non-Participant Survey (N = 152)**		
	**N**	**%**	**N**	**%**	**p-value**

Specialty					
Family Practice	208	39%	62	44%	0.2074
Pediatrics	312	58%	73	51%	
Other	15	3%	7	5%	
Practice ownership/affiliation					
Private independent office (solo or group)	454	76%	102	67%	0.0162
University or hospital medical center	73	12%	26	17%	
Physician network	33	5%	17	11%	
Other	41	7%	7	5%	
Proportion of pediatric patients covered by Medicaid					
Less than 10%	126	21%	29	19%	0.8415
10–50%	334	56%	88	59%	
More than 50%	134	23%	33	22%	
No. of vaccines given to children in a typical week					
Less than 10 vaccines/week	89	15%	30	20%	0.4930
10–25 vaccines/week	105	18%	26	17%	
26–100 vaccines/week	213	36%	48	32%	
More than 100 vaccines/week	185	31%	46	31%	
No. of physicians in practice giving childhood vaccines					
1–2	331	55%	85	57%	0.6628
3 or more	266	45%	63	43%	

**Table 2:** Reasons Given by Registry Non-Participants for Not Participating (N = 95)

	Reason for Not Participating*	
	
	**N**	**%**
Too much cost/staff time to participate	36	38%
Practice has own system for recording and monitoring immunizations	35	37%
Practice has not yet been recruited for or told about the registry**	21	22%
Registry not compatible with practice's computer system	20	21%
Confidentiality concerns	14	15%
Small number of pediatric patients	12	13%
Insufficient technical assistance available	9	9%

* Respondents were directed to choose all responses that applied.

** This response was written in by respondents as an "other" reason.

**Table 3:** Influence of Factors on Decision to Participate in Registry (N = 374)

	**Very Influential**	**Somewhat Influential**	**Not Influential**
Need to consolidate records for patients who receive vaccines at multiple sites	63%	28%	9%
State mandates participation*	54%	26%	20%
Nursing/administrative staff in favor of participation	40%	35%	25%
Ability to use registry to monitor immunization rates	33%	36%	31%
Medicaid/health plan mandates participation	32%	31%	37%
Compatibility of registry technology with office computers	27%	37%	36%
Availability of technical assistance/training from state	25%	41%	34%
Expected cost/staff time required for data input	21%	45%	34%

* This option given only in states with an existing legislative mandate.

**Table 4:** Problems Experienced by Registry Participants (N = 374)

	**Not a problem**	**A small problem**	**A significant problem**
Cost/staff time associated with using registry	44%	36%	20%
Concerns with accuracy or completeness of data	47%	37%	16%
Difficulty in accessing registry (e.g., busy phone lines)	58%	30%	12%
Problems with software/computer compatibility	62%	23%	15%
Inadequate technical support or training	73%	18%	9%
Confidentiality concerns	82%	13%	5%
