# Supplementary material for: Private provider participation in statewide immunization registries
Source: BMC Public Health. 2006 Feb 15;6:33. doi: 10.1186/1471-2458-6-33 (PMC1382205; doi:10.1186/1471-2458-6-33)
Supplement: Additional File 1 — Templates of registry participant and registry non-participant survey instruments. [file 1471-2458-6-33-S1.pdf]

VACCINE PROVIDER PERCEPTIONS OF THE [*insert name of state registry*]

This brief survey explores barriers to and influences on vaccine providers' participation in the [*insert name of state registry*]. All responses will be kept confidential. To complete the survey, please circle the number of your response, unless otherwise directed.

1. Do you (or does your practice) currently participate in [*insert state registry name*]?

1 No 2 Unsure 3 Yes ⇒ ***GO TO Question 6 on back***

2. Have you/your practice ever been contacted about participating in [*insert state registry name*]?

1 No → (go to Question 3)
2 Unsure → (go to Question 3)
3 Yes →

2a. Who contacted you? (*circle all that apply*)
1 State immunization program staff
2 Managed care organization
3 Other physicians/administrators in practice
4 Professional association
5 Other ___________________________________

2b. How were you contacted? (*circle all that apply*)
1 Letter
2 Personal contact in practice
3 Meeting/conference
4 Other ___________________________________

3. In your opinion, why does your practice <u>not participate</u> in [*insert state registry name*]? *(circle all that apply)*
1 Registry not compatible with practice's computer system
2 Too much staff time / cost to participate
3 Confidentiality concerns
4 Small number of pediatric patients
5 Practice has its own system for recording and monitoring immunizations
6 Insufficient technical assistance available
7 Other (*specify*) ____

____

4. Which of the following would be <u>essential</u> to gaining your participation in [*insert state registry name*]? ***(circle all that apply)***

1 Automated data entry
2 Technology compatible with office computers
3 On-site technical assistance from registry staff
4 Support for registry from state medical/specialty society
5 More support for participation from practice's nursing/administrative staff
6 State law mandating private practice participation in the registry [*option only in states with existing mandate*]
7 Legal advice/expertise to address confidentiality concerns
8 Increased participation among other vaccine providers in my community

5. On a scale of 0 to 10, how likely are you to begin participating in [*insert state registry name*] in the next two years? _______

6. How important to your practice are the following potential benefits of an immunization registry?

	Not Important	*Somewhat Important*	*Very Important*
1 Easy access to records of vaccines provided to my patients at other sites	1	2	3
2 Ability to generate reminder/recall notices	1	2	3
3 Ability to assess practice's immunization coverage	1	2	3
4 Printable immunization records for patients	1	2	3
5 Ability to document vaccines given for HEDIS, managed care organizations	1	2	3

7. In the past 2 years, have you received information regarding your practice's childhood immunization rate?

1 No   2 Unsure   3 Yes ⇒ **7a. What was data source for this information?**

1 Internal audit using practice records
2 HEDIS, other external audit
3 Internal audit using registry data
4 Health department assessment
5 Other ___________________________

8. What is the most important thing registry officials could do to increase/improve provider participation in [*insert state registry name*]?

____

____

____

Please tell us about your practice.

9. What is your specialty?

1 Pediatrics   2 Family Practice   3 Other_______________

10. How many physicians in your practice provide childhood vaccines? ___________

11. In a typical week, how many vaccines are administered to children?

1 <10 vaccines/wk   2 10-25 vaccines/wk   3 26-100 vaccines/wk   4 >100 vaccines/wk

12. What proportion of your practice's pediatric patients are covered by Medicaid (including fee-for-service and managed care Medicaid plans)?

1 None   2 Less than 10%   3 10%-50%   4 More than 50%

13. What is the practice's ownership/affiliation?

1 University or hospital medical center
2 Private, independent office
3 Managed care organization
4 Physician network
5 Public clinic
6 Other (*specify)*_________________________________

Thank you for your time. Please return this survey in the prepaid, addressed envelope provided.

VACCINE PROVIDER UTILIZATION OF THE [*insert state registry name]*

This brief survey explores benefits, barriers, and influences on vaccine providers' participation in the [*insert state registry name*]. All responses will be kept confidential. To complete the survey, please circle the number of your response, unless otherwise directed.

1. Do you (or does your practice) currently participate in [*insert state registry name*]?

1 Yes 2 Unsure 3 No ⇒ ***GO TO Question 10 on back***

2. How long have you participated in [*insert state registry name*]?

1 Less than 1 year 2 1 to 2 years 3 More than 3 years 4 Unsure

3. How do you use [*insert state registry name*] in your practice? (*circle all that apply*)

1 Input data on vaccines given
2 Review immunization records of individual patients
3 Print immunization records for patients
4 Generate reminder/recall notices for patients due for vaccines
5 Assess immunization coverage for practice
6 Other (*specify*) ______________________________
7 Not actively using registry

4. How influential were the following factors in your practice's decision to participate in [*state registry name*]?

	Not Influential	***Somewhat Influential***	***Very Influential***
1 Need to consolidate records for patients who receive vaccines at multiple sites	1	2	3
2 Compatibility of registry technology with office computers	1	2	3
3 Ability to use registry to monitor immunization rates	1	2	3
4 State law mandating registry participation [*option only in states with existing mandate*]	1	2	3
5 Medicaid/health plan mandates participation	1	2	3
6 Availability of technical assistance/training from state	1	2	3
7 Nursing/administrative staff in favor of participation	1	2	3
8 Expected cost/staff time required for data input	1	2	3

5. Overall, what was the <u>biggest</u> influence on your practice's decision to participate in [*state registry name*]?

____

____

6. Which staff are responsible for reporting your practice's immunization data to [*insert state registry name*]?

1 Nurses
2 Clerical/billing staff
3 Other (*specify*) ______________________________
4 NA – Data not reported ⇒ ***SKIP TO Question 9***

7. On average, how many hours per week does staff spend reporting data to [*insert state registry name*]?

1 Less than 2 hrs/wk 2 2-5 hrs/wk 3 5-10 hrs/wk 4 >10 hrs/wk 5 Unsure

8. How does your practice interact with [*insert state registry name*] to input and retrieve data?

1 Via internet connection
3 Hard copy of data sent by mail or fax
2 Via modem
4 Other (*specify*) ______________________________

9. To what extent has your practice experienced any of the following problems with [*insert state registry name*]?

	Not a problem	*A small problem*	*A significant problem*
1 Cost/staff time associated with using registry	1	2	3
2 Problems with software/computer compatibility	1	2	3
3 Difficulty in accessing registry (e.g., busy phone lines)	1	2	3
4 Concerns with accuracy or completeness of data	1	2	3
5 Confidentiality concerns	1	2	3
6 Inadequate technical support or training	1	2	3
7 Other (*specify*) ___________________________________	1	2	3

10. What is the <u>most important</u> thing registry officials could do to increase/improve provider participation in [*insert state registry name*]?

____

____

____

Please tell us about your practice.

11. What is your specialty?

1 Pediatrics 2 Family Practice 3 Other__________________

12. How many physicians in your practice provide childhood vaccines? ___________

13. In a typical week, how many vaccines are administered to children?

1 <10 vaccines/wk 2 10-25 vaccines/wk 3 26-100 vaccines/wk 4 >100 vaccines/wk

14. What proportion of your practice's pediatric patients are covered by Medicaid (including fee-for-service and managed care Medicaid plans)?

1 None 2 Less than 10% 3 10%-50% 4 More than 50%

15. What is the practice's ownership/affiliation?

1 University or hospital medical center
4 Physician network
2 Private, independent office
5 Public clinic
3 Managed care organization
6 Other (*specify)*________________________________

Thank you for your time. Please return this survey in the prepaid envelope provided.
